# Supplementary material for: Mammals show faster recovery from capture and tagging in human-disturbed landscapes
Source: Nat Commun. 2024 Sep 15;15:8079. doi: 10.1038/s41467-024-52381-8 (PMC11402999; doi:10.1038/s41467-024-52381-8)
Supplement: Supplementary file 1 — Supplementary Information [file 41467_2024_52381_MOESM1_ESM.pdf]

Supplementary Information

Mammals show faster recovery from capture and tagging in human-disturbed landscapes

Species table

Tab. S1: Overview of mammal species included in this study, the respective sample sizes, body mass, and dietary type.

**Family**	**Genus**	**Species**	$\mathbf{n_{ACC}}^{\alpha}$	$\mathbf{n_{GPS}}^{\alpha}$	**Mass [g]**$^{\beta}$	**Diet**
Felidae	*Acinonyx*	*jubatus*	0	14	46700	carnivore
Cervidae	*Alces*	*alces*	69	63	356998	herbivore
Bovidae	*Antidorcas*	*marsupialis*	7	10	31500	herbivore
Bovidae	*Bison*	*bison*	13	0	579255	herbivore
Bovidae	*Bison*	*bonasus*	27	26	500000	herbivore
Canidae	*Canis*	*aureus*	6	7	10345	omnivore
Canidae	*Canis*	*latrans*	18	0	13406	omnivore
Canidae	*Canis*	*lupus*	10	14	32183	carnivore
Bovidae	*Capra*	*ibex*	34	24	85167	herbivore
Cervidae	*Capreolus*	*capreolus*	672	547	22500	herbivore
Cervidae	*Cervus*	*elaphus*	34	43	131250	herbivore
Cercopithecidae	*Chlorocebus*	*pygerythrus*	12	12	3975	omnivore
Hyaenidae	*Crocuta*	*crocuta*	7	6	63000	carnivore
Eupleridae	*Cryptoprocta*	*ferox*	11	4	9500	carnivore
Equidae	*Equus*	*hemionus hemionus*	14	60	230000	herbivore
Erinaceidae	*Erinaceus*	*europaeus*	12	12	771	carnivore
Lemuridae	*Eulemur*	*rufifrons*	7	6	1820	herbivore
Felidae	*Felis*	*chaus*	12	12	7393	carnivore
Felidae	*Felis*	*silvestris*	5	5	5500	carnivore
Bovidae	*Gazella*	*subgutturosa*	7	7	28500	herbivore
Viverridae	*Genetta*	*genetta*	7	5	1800	carnivore
Herpestidae	*Ichneumia*	*albicauda*	6	5	3500	omnivore
Leporidae	*Lepus*	*europaeus*	63	47	3740	herbivore
Felidae	*Lynx*	*lynx*	10	6	17950	carnivore
Felidae	*Lynx*	*rufus*	35	35	8904	carnivore
Bovidae	*Madoqua*	*guentheri*	6	6	7500	herbivore
Cervidae	*Odocoileus*	*virginianus*	5	9	55509	herbivore
Bovidae	*Ovibos*	*moschatus*	6	6	340501	herbivore
Felidae	*Panthera*	*leo*	14	12	161499	carnivore
Felidae	*Panthera*	*pardus*	3	3	55000	carnivore
Cercopithecidae	*Papio*	*anubis*	6	6	28329	omnivore
Procyonidae	*Procyon*	*lotor*	12	12	6550	omnivore
Indriidae	*Propithecus*	*verreauxi*	14	14	3250	herbivore
Felidae	*Puma*	*concolor*	13	12	51600	carnivore
Suidae	*Sus*	*scrofa*	159	98	101052	omnivore
Bovidae	*Tragelaphus*	*oryx*	8	8	569994	herbivore
Bovidae	*Tragelaphus*	*strepsiceros*	7	7	213501	herbivore
Ursidae	*Ursus*	*americanus*	42	46	99949	omnivore
Ursidae	*Ursus*	*arctos*	14	15	180520	omnivore
Viverridae	*Viverra*	*tangalunga*	18	12	6885	omnivore
Canidae	*Vulpes*	*bengalensis*	8	8	2726	omnivore
Canidae	*Vulpes*	*vulpes*	19	18	5318	omnivore

$^{\alpha}$ only individuals with continuous data during the first 20 days of tracking were considered.

$^{\beta}$ data from trait database: Faurby et al. 2018, Phylacine 1.2: The Phylogenetic Atlas of Mammal Macroecology *Ecology* 99:2626. The mass values reported are rounded to the nearest gram.

Disturbance intensity - species plots

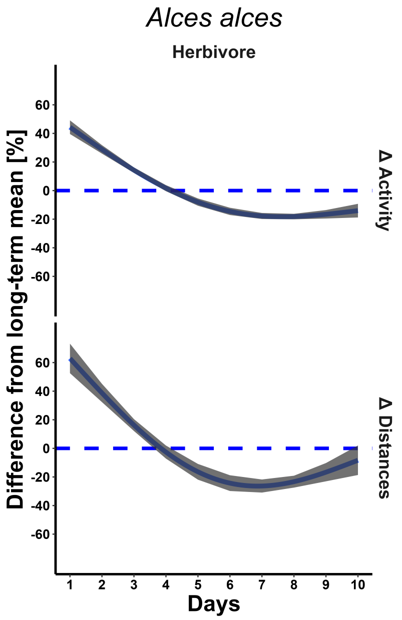

Fig. S1A: *Alces alces*

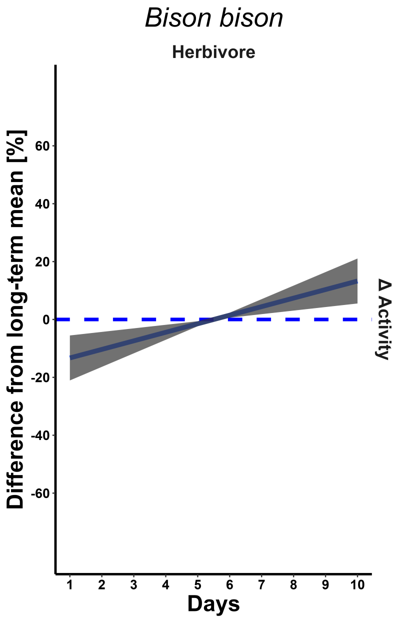

Fig. S1B: *Bison bison*

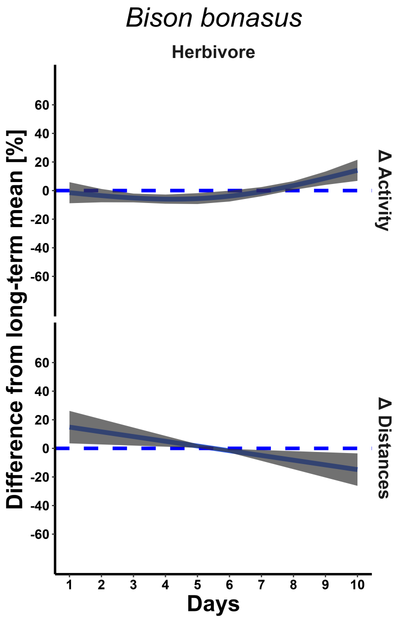

Fig. S1C: *Bison bonasus*

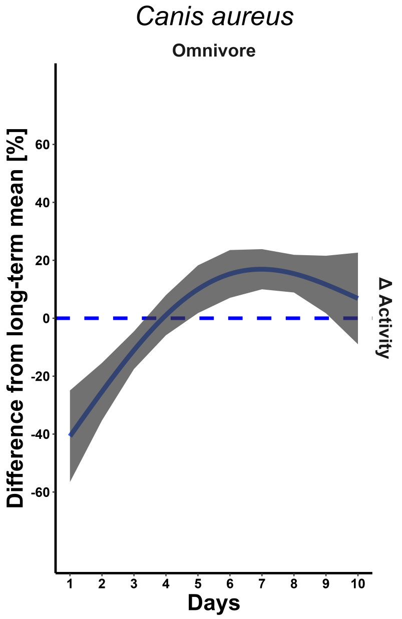

Fig. S1D: *Canis aureus*

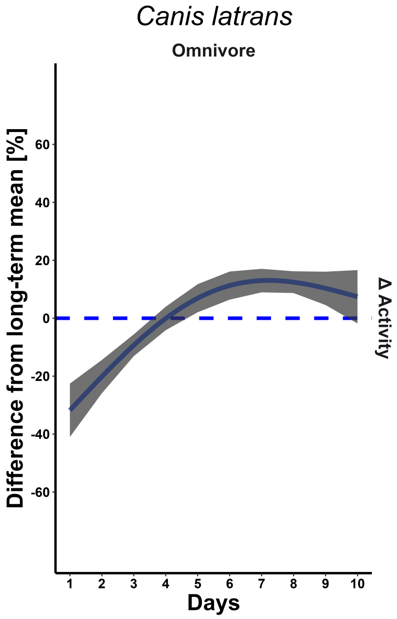

Fig. S1E: *Canis latrans*

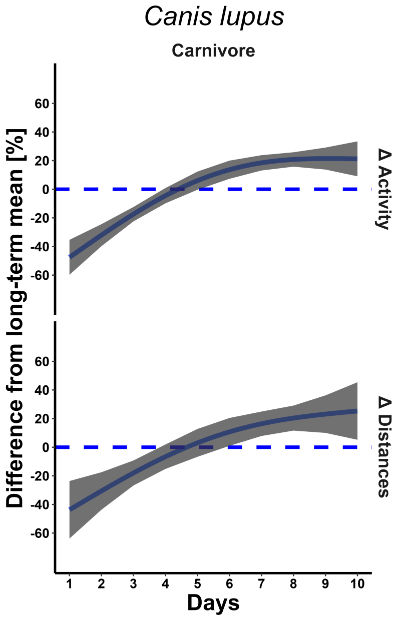

Fig. S1F: *Canis lupus*

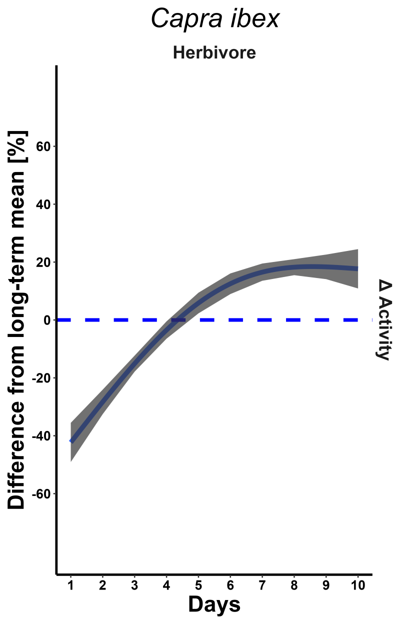

Fig. S1G: *Capra ibex*

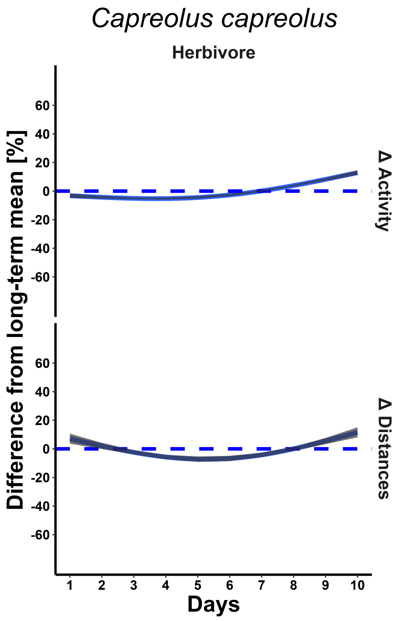

Fig. S1H: *Capreolus capreolus*

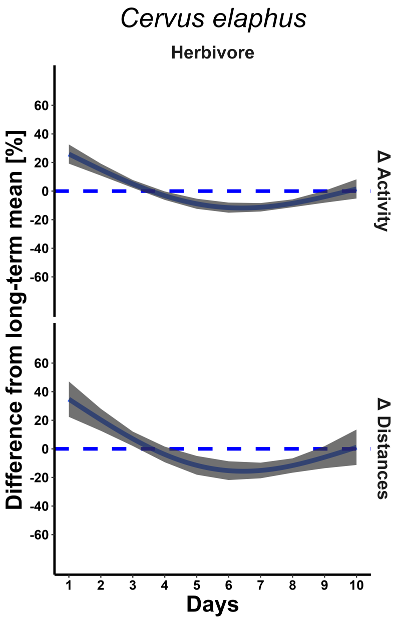

Fig. S1I: *Cervus elaphus*

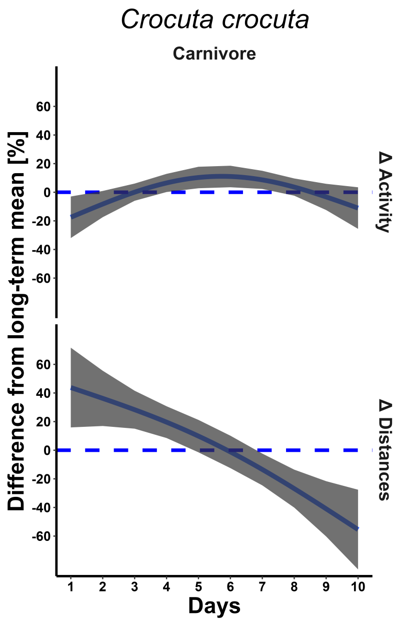

Fig. S1J: *Crocuta crocuta*

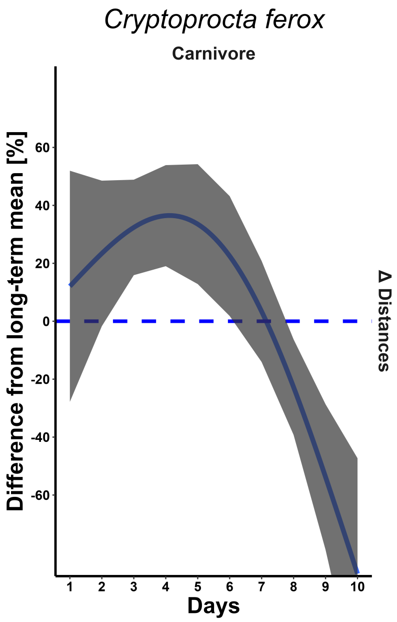

Fig. S1K: *Cryptoprocta ferox*

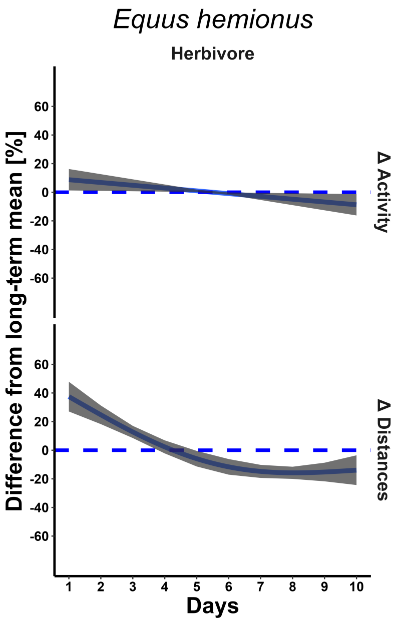

Fig. S1L: *Equus hemionus*

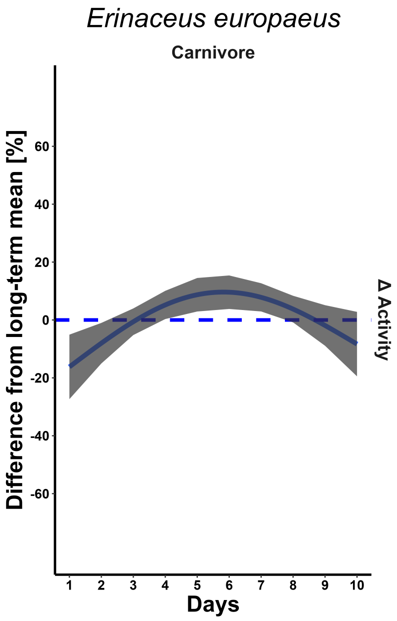

Fig. S1M: *Erinaceus europaeus*

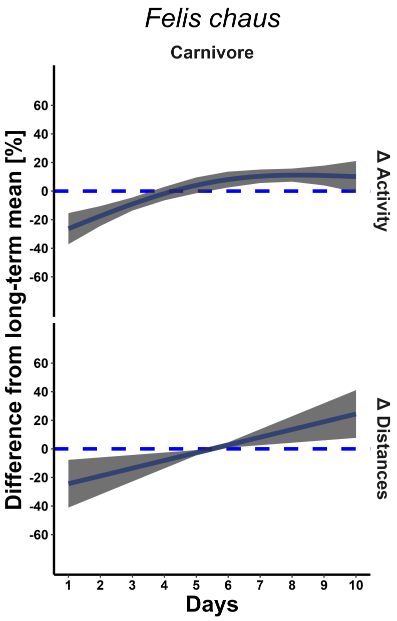

Fig. S1N: *Felis chaus*

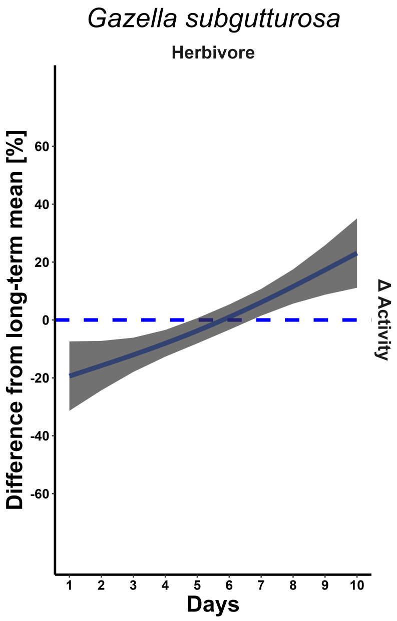

Fig. S1O: *Gazella subgutturosa*

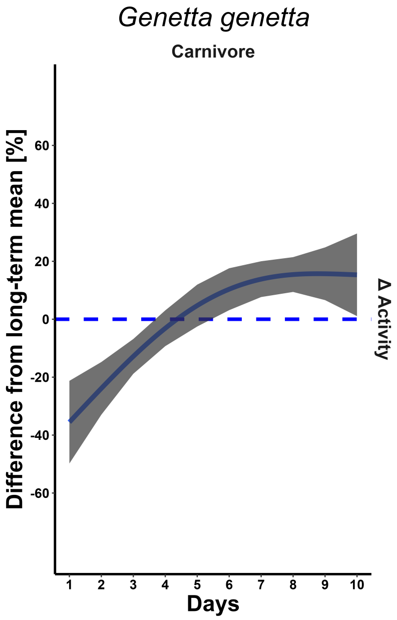

Fig. S1P: *Genetta genetta*

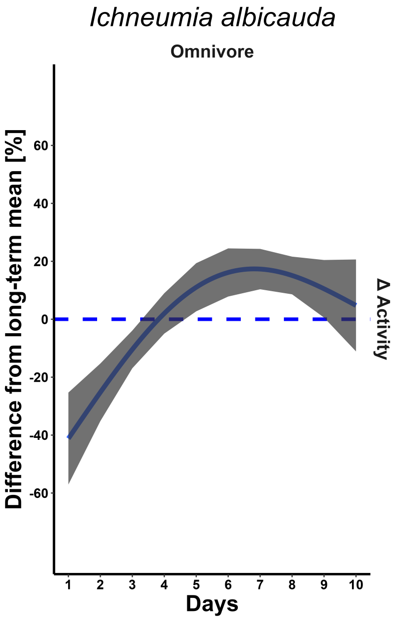

Fig. S1Q: *Ichneumia albicauda*

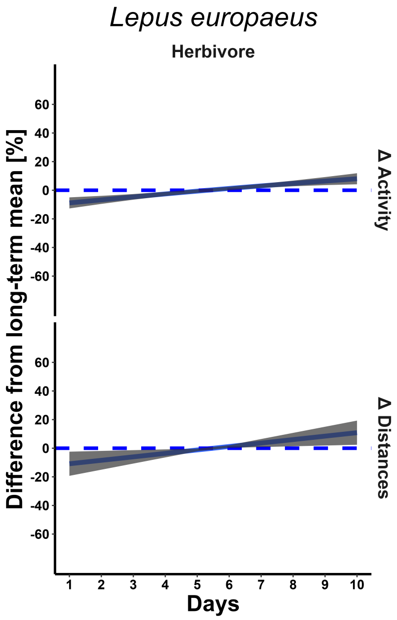

Fig. S1R: *Lepus europaeus*

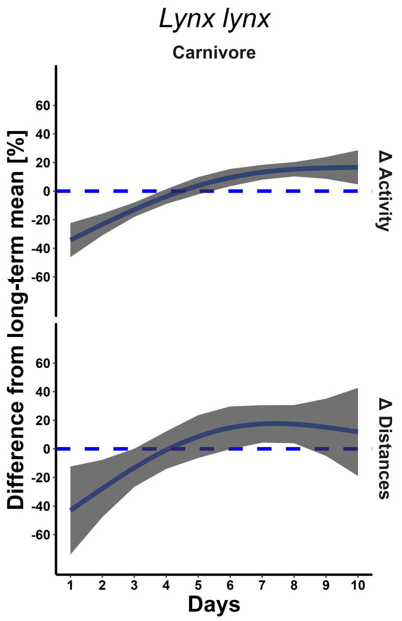

Fig. S1S: *Lynx lynx*

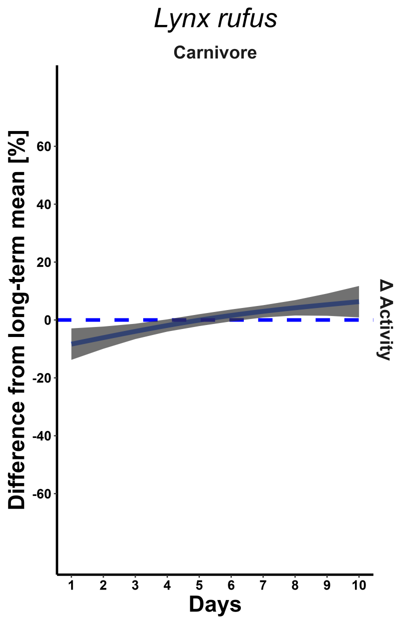

Fig. S1T: *Lynx rufus*

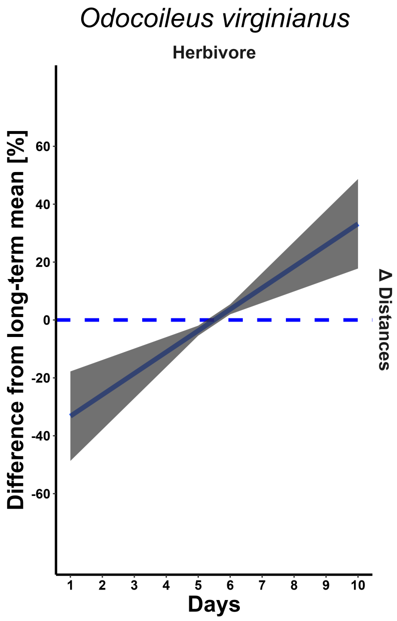

Fig. S1U: *Odocoileus virginianus*

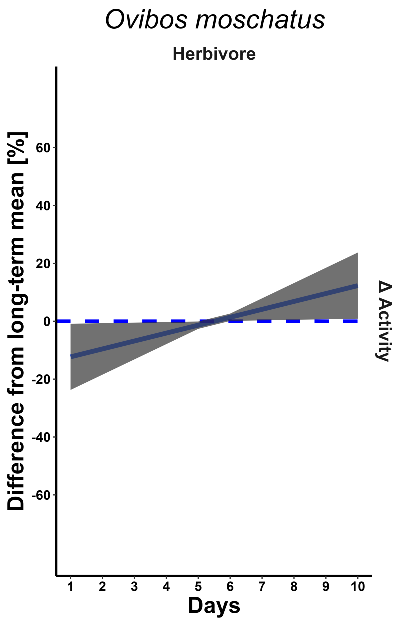

Fig. S1V: *Ovibos moschatus*

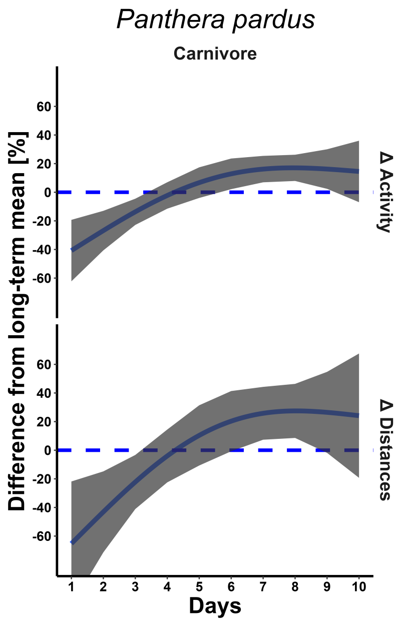

Fig. S1W: *Panthera pardus*

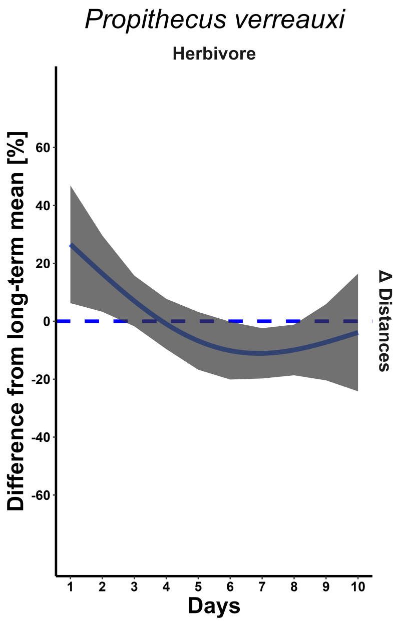

Fig. S1X: *Propithecus verreauxi*

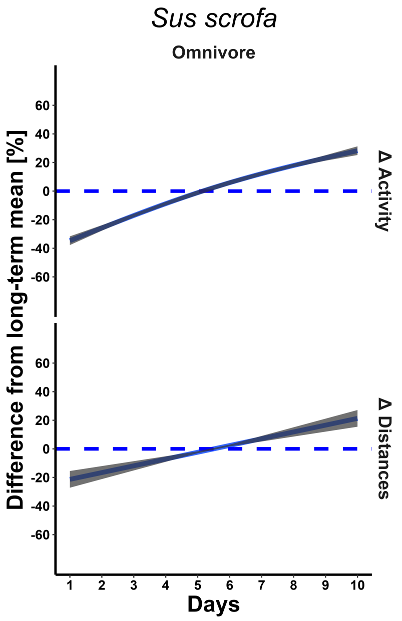

Fig. S1Y: *Sus scrofa*

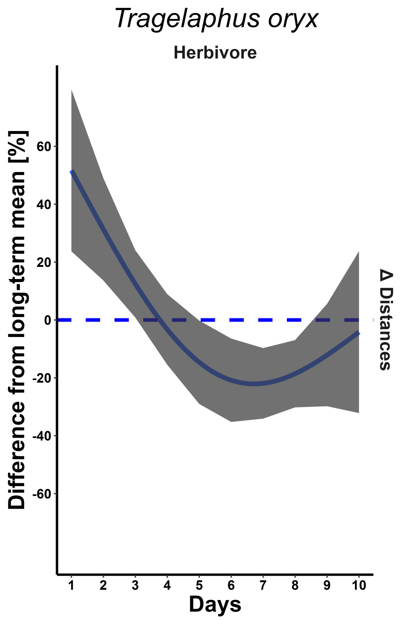

Fig. S1Z: *Tragelaphus oryx*

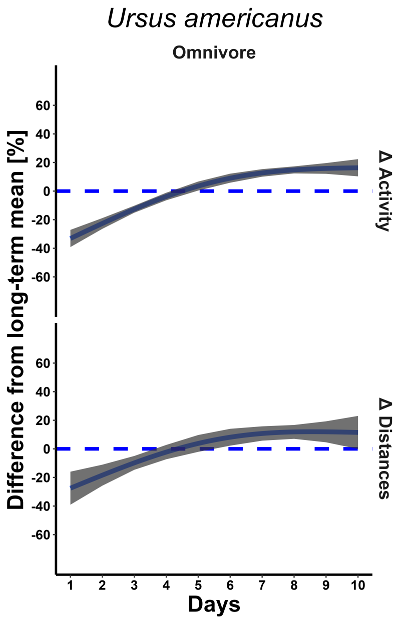

Fig. S1AA: *Ursus americanus*

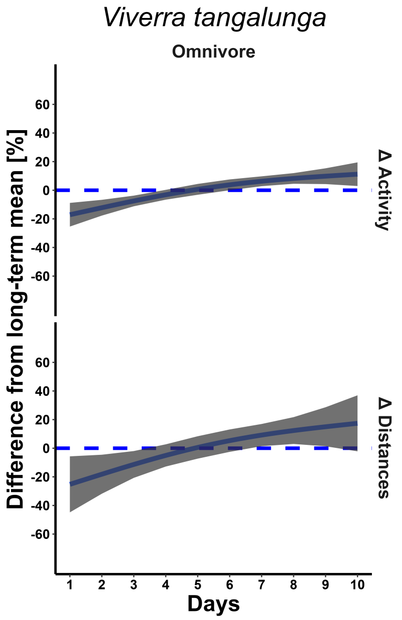

Fig. S1AB: *Viverra tangalunga*

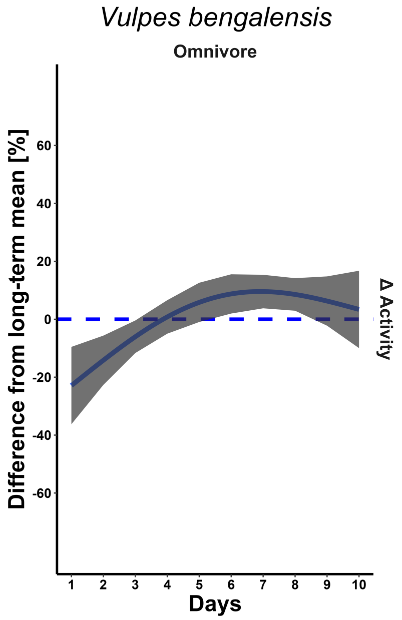

Fig. S1AC: *Vulpes bengalensis*

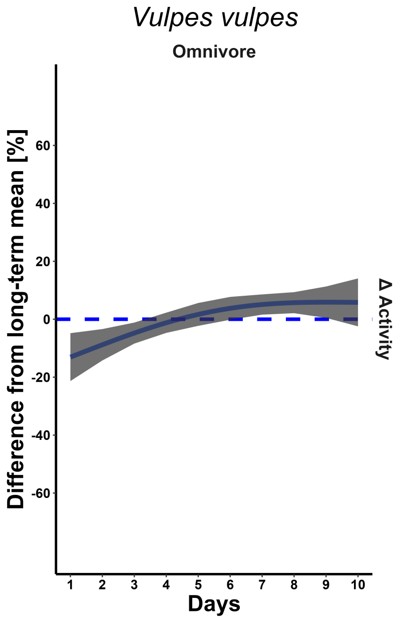

Fig. S1AD: *Vulpes vulpes*

Fig. S1A-S1AD: Disturbance intensity: Impacts of collaring on activity (upper) and displacements (lower) during the initial days after release. Daily differences to the long-term mean of displacements and/or activity spent for all species with $p < 0.05$; $modeled\ value \pm SE$ (Tab. 1); $n_{Activity} = 1241$, $n_{Displacements} = 1014$.

Model selection tables

Tab. S2: Model selection table Activity

Model	(Intercept)	diet	mass	Hfi	sex	df	logLik	AICc	delta	weight
6	-2.304	+		1.732		6	-2236.65	4485.4	0	0.221
14	-2.545	+		1.787	+	7	-2235.781	4485.7	0.28	0.192
7	-5.463		0.367	1.648		5	-2238.205	4486.5	1.09	0.128
15	-5.957		0.3934	1.706	+	6	-2237.323	4486.7	1.35	0.113
8	-5.101	+	0.2893	1.776		7	-2236.381	4486.9	1.49	0.105
16	-5.51	+	0.3086	1.827	+	8	-2235.415	4486.9	1.58	0.101
5	-1.491			1.547		4	-2239.672	4487.4	2.01	0.081
13	-1.755			1.621	+	5	-2238.997	4488	2.68	0.058
1	2.355					3	-2248.989	4504	18.63	0
9	2.294				+	4	-2249.142	4506.3	20.95	0
2	2.38	+				5	-2249.875	4509.8	24.43	0
3	2.242		0.01124			4	-2250.95	4509.9	24.57	0
11	2.078		0.02119		+	5	-2251.04	4512.1	26.76	0
10	2.311	+			+	6	-2250.08	4512.2	26.86	0
4	2.086	+	0.03275			6	-2251.576	4515.2	29.85	0
12	1.929	+	0.04197		+	7	-2251.69	4517.5	32.1	0

Tab. S3: Model selection table Displacements

Model	(Intercept)	diet	mass	HFi	sex	df	logLik	AICc	delta	weight
4	0.6951	+	0.2508			6	-1584.3	3180.7	0	0.475
3	0.5377		0.2262			4	-1586.66	3181.4	0.68	0.338
8	0.5844	+	0.2585		+	7	-1585.31	3184.7	4.05	0.063
1	3.011					3	-1589.58	3185.2	4.5	0.05
7	0.4307		0.233		+	5	-1587.8	3185.7	4.97	0.04
2	3.291	+				5	-1588.68	3187.4	6.74	0.016
5	2.982				+	4	-1590.86	3189.8	9.08	0.005
12	0.8163	+	0.2447	-0.00336		7	-1587.87	3189.8	9.16	0.005
11	0.7087		0.217	-0.0049		5	-1590.14	3190.3	9.67	0.004
6	3.266	+			+	6	-1589.91	3191.9	11.23	0.002
9	3.157			-0.01042		4	-1592.74	3193.5	12.83	0.001
16	0.6585	+	0.2549	-0.00204	+	8	-1588.9	3193.9	13.26	0.001
15	0.5628		0.2259	-0.0037	+	6	-1591.31	3194.7	14.03	0
10	3.442	+		-0.00962		6	-1591.88	3195.8	15.16	0
13	3.117			-0.00939	+	5	-1594.08	3198.2	17.54	0
14	3.401	+		-0.00851	+	7	-1593.17	3200.4	19.77	0

Supplementary Note 1

Animal Tracking Permits

Acinonyx jubatus: All experimental procedures described were approved by the Internal Ethics Committee of the Leibniz Institute for Zoo and Wildlife Research (Leibniz-IZW, Berlin, Germany) (permit number: 1 April 2002) and the Ministry of Environment, Forestry and Tourism of Namibia (permit numbers: 1689/2012, 1813/2013, 1914/2014, 2067/2015, 2194/2016, 2208/2017, RCIV00082018/2018050101).

Alces alces: The capture and handling of GPS-marked moose in Norway was approved by the Norwegian Environment Agency (capture) and the Norwegian Food Safety Authority, which is the Norwegian animal research authority (permits no. 16/258650, 07/68902 and 2015/232016).

Antidorcas marsupialis: Approved by the Namibian Council on Research, Science and Technology, certificate: RCIV00032018.

Genetta genetta: Approved by The National Council of Science Technology and Innovation (permit number NACOSTI/P/14/357/2062), Kenya Wildlife Service (permit number KWS/BRM/5001) and through the Smithsonian Institution's National Museum of Natural History's Animal Care and Use Committee (Animal Study Proposal 2014-11).

Bison bison: Approved by the Nature Conservancy (Missouri) and Missouri Department of Natural Resources.

Bison bonasus: This study was carried out under research permits no. DLOPiK-op/ogiz-4200/IV.A-38-1/8310,10568/07/wo from the Polish Ministry of Environment and no. DOPozgiz-4200/IV.A-4/208/10/ls from the General Director for Environmental Protection in Poland, as well as ethics, permits no. 31/2006, and 2009/52 from the Local Ethical Commission in Białystok, Poland.

Canis aureus: All captures and handling were approved by the Maharashtra State Forest Department: permit no. SPP-147, dated 17.3.2015.

Canis latrans: Approved by Mississippi State University Institutional Animal Care and Use Committee, protocols 09-004, 12-012.

Canis lupus: Approved by Mississippi State University Institutional Animal Care and Use Committee, protocols 09-004, 12-012.

Capra ibex: The body in charge of the Alpine ibex capture was the Gran Paradiso National Park. Ibex capture and handling protocols were approved by the Italian Ministry of Environment (Protoc. no. 25114/04).

Capreolus capreolus: (IZW-Berlin) Approved by the "LUGV Brandenburg", permit: 23-2347-1-2009.

Capreolus capreolus: Game captures were conducted in accordance with European and French laws. The experiment was designed to minimize animal stress and handling time and to ensure animal welfare, as defined in guidelines for the ethical use of animals in research. A specific accreditation was also delivered to the OFB for capturing animals for scientific and wildlife management purposes. All methods were approved by the authorities (French Ministry of Environment). Roe deer captures and experimental procedures were in line with the French Environmental Code (Art.R421-15 to 421-31 and R422-92 to 422-94-1) and duly approved by legislation from the Prefecture of Paris (Prefectural Decree no. 2009-014).

Capreolus capreolus: All capture and marking procedures were done in accordance with French and European laws for animal welfare (prefectural order from the Toulouse Administrative Authority to capture and monitor wild roe deer and agreement no. A31113001 approved by the Departmental Authority of Population Protection).

Capreolus capreolus: Permit provided by the government of Upper Bavaria (ROB-55.2Vet-2532.Vet 02-17-190).

Capreolus capreolus: Resolution of the Provincial Government n. 602, under approval of the Wildlife Committee of 20/09/2011, and successive integration approved on the 23/04/2015.

Capreolus capreolus: The animal capture and handling protocols were authorized by the cantonal veterinary and animal welfare services with permit number BE75/11.

Cervus elaphus: Game captures were conducted in accordance with European and French laws. The experiment was designed to minimize animal stress and handling time and to ensure animal welfare, as defined in guidelines for the ethical use of animals in research. A specific accreditation was also delivered to the OFB for capturing animals for scientific and wildlife management purposes. Red deer captures, and experimental procedures were in line with the French Environmental Code (Art.R421-15 to 421-31 and R422-92 to 422-94-1) and duly approved by legislation from the Prefecture of Paris (Prefectural Decree no. 2009-014 and no. 2015-020).

Cervus elaphus: Permit provided by the Ministry of the Environment of the Czech Republic, number MZP/2019/630/361.

Cervus elaphus: Permit provided by the government of Upper Bavaria (Az. 55.2-1-54-2531-89-09).

Chlorocebus pygerythrus: The study was conducted with permission from the Kenya Government (NACOSTI permit no. P/15/5820/4650) and under IACUC protocol no. 17477 from the University of California, Davis.

Crocuta crocuta: Animal handling protocols were approved and conducted with the ethical clearance of the Animal Research Ethics Committee of the University of KwaZulu-Natal, South Africa (009/13/Animal), and the Institutional Animal Care and Use Committee of University of California at Berkeley (IACUC Protocol #R217-0512B) and Virginia Tech (IACUC Protocol #15-012). Scientific collecting permits were authorized from the Ministry of Environment and Tourism, Namibia (Research/Collecting Permits 1724/2012, 1834/2013,

1956/2014) and from the Department of Wildlife and National Parks, Botswana (Research Permit EWT 8/36/4 XXVIII [35]).
Cryptoprocta ferox: All research protocols were approved by the appropriate animal Use and care committees of Germany (”Bundesministerium für Naturschutz, BfN”) and Madagascar (”Ministère de l'Environnement et des Eaux et Forêts, MINEEF”).

Equus hemionus: Animal tracking permit provided by the Ministry of Environment and Tourism, Mongolia.

Erinaceus europaeus: (IZW-Berlin) Approved by ethical standards of the institution (IZW permit 2016-02-01), German law ”Tierversuchsgenehmigung” permission numbers: Reg0115/15, and G0104/14, and the local nature conservation authority.

Eulemur rufifrons: All research protocols were approved by the appropriate Animal Use and Care Committees of Madagascar (Ministère de l'Environnement et des Eaux et Forêts,
MINEEF: No 90/16/MEEMF/SG/DGF/DAPT/SCBT.RE, No 72/17/MEEMF/SG/DGF/DSAP/SCBT.RE.

Felis chaus: All captures and handling were approved by the Maharashtra State Forest Department: permit no. SPP-147, dated 17.3.2015.

Felis silvestris: Approved by the local nature conservation authority, permit number: ASTURIAS 2018/002528 LEON EP/CYL/666/2018.

Gazella subgutturosa: Animal tracking permit provided by the Ministry of Environment and Tourism, Mongolia.

Genetta genetta: Approved by The National Council of Science Technology and Innovation (permit number NACOSTI/P/14/357/2062), Kenya Wildlife Service (permit number KWS/BRM/5001) and through the Smithsonian Institution's National Museum of Natural History's Animal Care and Use Committee (Animal Study Proposal 2014-11).

Ichneumia albicauda: Approved by The National Council of Science Technology and Innovation (permit number NACOSTI/P/14/357/2062), Kenya Wildlife Service (permit number KWS/BRM/5001) and through the Smithsonian Institution's National Museum of Natural History's Animal Care and Use Committee (Animal Study Proposal 2014-11).

Lepus europaeus: Animal tracking was obtained in accordance with the Federal Nature Conservation Act (§ 45 Abs. 7 Nr. 3) and approved by the local nature conservation authority (reference numbers: 2347-6-2019, LUGV V3- 2347-22-2013, and 55.2-1-54-2532-229-13).

Lynx lynx: Approved by the PLA Moravian Karst Administration and the Czech Ministry of Environment, permit numbers: SR/0081/JM/2017; 34128/ENV/17-2146/630/17). Lynx live-trapping in Poland was approved

by the National Ethics Committee for Animal Experiments (no. DB/KKE/PL—110/2001) and the Local Ethics Committee for Animal Experiments at the Medical University of Białystok, Poland (no. 52/2007).

Lynx lynx: Permit provided by the government of Upper Bavaria (Az. 55.2-1-54-2531-89-09).

Lynx rufus: All animal capture, handling, collaring, and sample collection was approved by the Institutional Animal Care and Use Committee (IACUC) of the University of California, Santa Cruz (Protocols ”Seril 1701”, and ”Seril 1701 a1”). Scientific collecting permits were authorized by the California Department of Fish and Wildlife (Aromas, SCP-11968; Coyote Valley, SCP-13565).

Lynx rufus: Approved by Mississippi State University Institutional Animal Care and Use Committee, protocols 09-004, 12-012.

Madoqua guentheri: The research permit was approved by Mpala Research Center, Laikipia, Kenya.

Odocoileus virginianus: All activities were conducted according to guidelines established by the American Society of Mammalogists, and with authorization from the Oklahoma Department of Wildlife Conservation.

Ovibos moschatus: The study was approved by the Government of Greenland (permit no. 2019-88).

Panthera leo: Animal handling protocols were approved and conducted with the ethical clearance of the Animal Research Ethics Committee of the University of KwaZulu-Natal, South Africa (009/13/Animal), and the Institutional Animal Care and Use Committee of University of California at Berkeley (IACUC Protocol #R217-0512B) and Virginia Tech (IACUC Protocol #15-012). Scientific collecting permits were authorized from the Ministry of Environment and Tourism, Namibia (Research/Collecting Permits 1724/2012, 1834/2013, 1956/2014) and from the Department of Wildlife and National Parks, Botswana (Research Permit EWT 8/36/4 XXVIII [35]).

Panthera pardus: The study was conducted with permission from the Kenya Government (NACOSTI permit no. P/15/5820/4650) and under IACUC protocol no. 17477 from the University of California, Davis.

Papio anubis: The study was conducted with permission from the Kenya Government (NACOSTI permit no. P/15/5820/4650) and under IACUC protocol no. 17477 from the University of California, Davis.

Propithecus verreauxi: All research protocols were approved by the appropriate Animal Use and Care Committees of Madagascar (Ministère de l’Environnement et des Eaux et Forêts, MINEEF: No 90/16/MEEMF/SG/DGF/ DAPT/SCBT.RE, No 72/17/MEEMF/SG/DGF/DSAP/SCBT.RE.

Procyon lotor: Approved by the ”LUGV”, permit number: 2347-7-2020.

Puma concolor: Research was approved by UCSC IACUC, proposal code Wilmc1312, and conducted under permit #11968.

Sus scrofa: All activities were conducted according to guidelines established by the American Society of Mammalogists; as defined by the Oklahoma Feral Swine Control Act (O.S § 6-601), only Judas pigs with tracking collars were released, all others were euthanized by law.

Sus scrofa: Approved by the ethics committee of the Ministry of the Environment Czech Republic number MZP/2019/630/361.

Sus scrofa: Approved by the Tuscany Regional Administration (permit number 103/5936/152 - 13/03/2002) and the Arezzo Province Administration (permit number 144160/42-41-2013 30/07/2013).

Sus scrofa: Approved by the Foreste Casentinesi National Park, permit numbers 626 - 10/12/2015 and 969 - 28/12/2018.

Sus scrofa: Approved by Regione Autonoma della Sardegna, permit number: 4753-74 del 07/03/2017.

Sus scrofa: Approved by regional council Tübingen, according to animal welfare law § 8.1 of the Federal State Baden-Württemberg, permit number: WFS1/12.

Tragelaphus oryx: Approved by the Namibian Council on Research, Science and Technology, certificate: RCIV00032018.

Tragelaphus strepsiceros: Approved by the Namibian Council on Research, Science and Technology, certificate: RCIV00032018.

Ursus americanus: Approved by Mississippi State University Institutional Animal Care and Use Committee, protocols 09-004, 12-012.

Ursus arctos: All captures and handling were approved by the Polish authorities (no. DOPOZ.6401.08.2.2013.ls, DOP-OZ. 6401.08.2.2013.ls.1, DZP-WG.6401.08.8.2014.JRO), Polish ethical committee (no. 21/2013 and 101/2014) and Decree of Polish Ministry of Environment (Dz.Urz.M.Ś. 2017 poz. 2) and/or Slovak Ministry of Environment (MZP SR c. 3555/2012-2.2).

Viverra tangalunga: Approved by the Sabah Biodiversity Centre and the Sabah Wildlife Department, license ref.no: JKM/MBS.10000-2/2 JLD.6[8].

Vulpes bengalensis: All captures and handling were approved by the Maharashtra State Forest Department: permit no. SPP-147, dated 17.3.2015.

Vulpes vulpes: Approved by the Himachal Pradesh Forest Department: WLM/Research study/1259, dated 10/05/2019.

Vulpes vulpes: Approved by the "Landesamt für Umwelt, Gesundheit und Verbraucherschutz Brandenburg" LUGV, permit number: 2347-25-2015 and V3-2347 13-2011.

Vulpes vulpes: Approved by the animal welfare licensing committee of Berlin ("Landesamt für Gesundheit und Soziales" LaGeSo), permit number: G0211/15.
