# Supplementary material for: Mammals show faster recovery from capture and tagging in human-disturbed landscapes
Source: Nat Commun. 2024 Sep 15;15:8079. doi: 10.1038/s41467-024-52381-8 (PMC11402999; doi:10.1038/s41467-024-52381-8)
Supplement: Supplementary file 3 — Reporting Summary [file 41467_2024_52381_MOESM3_ESM.pdf]

nature portfolio

Corresponding author(s): Jonas Stiegler

Last updated by author(s): Jul 11, 2024

Reporting Summary

Nature Portfolio wishes to improve the reproducibility of the work that we publish. This form provides structure for consistency and transparency in reporting. For further information on Nature Portfolio policies, see our Editorial Policies and the Editorial Policy Checklist.

Statistics

For all statistical analyses, confirm that the following items are present in the figure legend, table legend, main text, or Methods section.

n/a	Confirmed	
☐	☒	The exact sample size (*n*) for each experimental group/condition, given as a discrete number and unit of measurement
☒	☐	A statement on whether measurements were taken from distinct samples or whether the same sample was measured repeatedly
☐	☒	The statistical test(s) used AND whether they are one- or two-sided<br>*Only common tests should be described solely by name; describe more complex techniques in the Methods section.*
☐	☒	A description of all covariates tested
☒	☐	A description of any assumptions or corrections, such as tests of normality and adjustment for multiple comparisons
☐	☒	A full description of the statistical parameters including central tendency (e.g. means) or other basic estimates (e.g. regression coefficient) AND variation (e.g. standard deviation) or associated estimates of uncertainty (e.g. confidence intervals)
☐	☒	For null hypothesis testing, the test statistic (e.g. *F*, *t*, *r*) with confidence intervals, effect sizes, degrees of freedom and *P* value noted<br>*Give P values as exact values whenever suitable.*
☒	☐	For Bayesian analysis, information on the choice of priors and Markov chain Monte Carlo settings
☐	☒	For hierarchical and complex designs, identification of the appropriate level for tests and full reporting of outcomes
☒	☐	Estimates of effect sizes (e.g. Cohen's *d*, Pearson's *r*), indicating how they were calculated

Our web collection on statistics for biologists contains articles on many of the points above.

Software and code

Policy information about availability of computer code

Data collection	No software was used for data collection. The tables with the tracking data were provided directly by the data providers or the data was made accessible via the Movebank data repository.
Data analysis	Data analysis was conducted in R (4.3.2) with help of the packages "move (4.2.4)", "moveACC" (0.1), "adehabitatLT (0.3.27)", "mgcv (1.9)", "lme4 (1.1-35.1)" and "MuMIn (1.47.5)".

For manuscripts utilizing custom algorithms or software that are central to the research but not yet described in published literature, software must be made available to editors and reviewers. We strongly encourage code deposition in a community repository (e.g. GitHub). See the Nature Portfolio guidelines for submitting code & software for further information.

Data

Policy information about availability of data

All manuscripts must include a data availability statement. This statement should provide the following information, where applicable:

- Accession codes, unique identifiers, or web links for publicly available datasets
- A description of any restrictions on data availability
- For clinical datasets or third party data, please ensure that the statement adheres to our policy

The datasets generated in this study to create the respective figures have been provided in the Source Data file. The GPS and acceleration datasets used and analyzed in this study are partially available in the Movebank Data Repository; data from Euromammals can be accessed by logging into their website or via a contact form, and additional data can be obtained from data providers upon request through the corresponding author.

Research involving human participants, their data, or biological material

Policy information about studies with human participants or human data. See also policy information about sex, gender (identity/presentation), and sexual orientation and race, ethnicity and racism.

Reporting on sex and gender	*Use the terms sex (biological attribute) and gender (shaped by social and cultural circumstances) carefully in order to avoid confusing both terms. Indicate if findings apply to only one sex or gender; describe whether sex and gender were considered in study design; whether sex and/or gender was determined based on self-reporting or assigned and methods used. Provide in the source data disaggregated sex and gender data, where this information has been collected, and if consent has been obtained for sharing of individual-level data; provide overall numbers in this Reporting Summary. Please state if this information has not been collected. Report sex- and gender-based analyses where performed, justify reasons for lack of sex- and gender-based analysis.*
Reporting on race, ethnicity, or other socially relevant groupings	*Please specify the socially constructed or socially relevant categorization variable(s) used in your manuscript and explain why they were used. Please note that such variables should not be used as proxies for other socially constructed/relevant variables (for example, race or ethnicity should not be used as a proxy for socioeconomic status). Provide clear definitions of the relevant terms used, how they were provided (by the participants/respondents, the researchers, or third parties), and the method(s) used to classify people into the different categories (e.g. self-report, census or administrative data, social media data, etc.) Please provide details about how you controlled for confounding variables in your analyses.*
Population characteristics	*Describe the covariate-relevant population characteristics of the human research participants (e.g. age, genotypic information, past and current diagnosis and treatment categories). If you filled out the behavioural & social sciences study design questions and have nothing to add here, write "See above."*
Recruitment	*Describe how participants were recruited. Outline any potential self-selection bias or other biases that may be present and how these are likely to impact results.*
Ethics oversight	*Identify the organization(s) that approved the study protocol.*

Note that full information on the approval of the study protocol must also be provided in the manuscript.

Field-specific reporting

Please select the one below that is the best fit for your research. If you are not sure, read the appropriate sections before making your selection.

☐ Life sciences ☐ Behavioural & social sciences ☒ Ecological, evolutionary & environmental sciences

For a reference copy of the document with all sections, see nature.com/documents/nr-reporting-summary-flat.pdf

Ecological, evolutionary & environmental sciences study design

All studies must disclose on these points even when the disclosure is negative.

Study description	We analyzed post-tagging effects on 1585 individuals of 42 terrestrial mammal species using collar-collected GPS and accelerometer data. Tracking data was collected from multiple data providers.
Research sample	Tracking data of 1585 individuals of 42 terrestrial mammal species. All animals were caught and fitted with GPS/ACC collars, except for Erinaceus europaeus, which were fitted with a little backpack including GPS and ACC sensor.
Sampling strategy	Sample size was determined by all tracking data of mammals obtainable, hence sample size differs across species.
Data collection	Data was collected by multiple data providers (co-authors).

Timing and spatial scale	We analyzed the initial 20 days of tracking data for each individual with continuous, non-missing data. We defined data as missing if any discontinuation resulted in less than 1 GPS fix per hour and less than one activity measurement per 30 minutes.
Data exclusions	tracking data of individuals was excluded, when missing data/discontinuation of data was present in the first 20 days post-release (see above).
Reproducibility	We applied Generalized Additive Mixed Models for "Disturbance intensity", and linear mixed effect models for "Recovery speed".
Randomization	Focal individuals were randomly chosen to be trapped and gps-collared depending on the specific study design (trapping / chasing).
Blinding	Each individual study pursued a specific research objective for its study. In this study, we used and analyzed the data for a different purpose, so blinding is not applicable.

Did the study involve field work? ☒ Yes ☐ No

Field work, collection and transport

Field conditions	Fieldwork was done across different continents and seasons, hence field conditions are related to the individual tracking studies included in our study.
Location	see above.
Access & import/export	see above, for permits see "Ethics oversight".
Disturbance	Animals were caught, temporary restrained, fitted with GPS-collars, and released. Trapping was done differently across study species and studies.

Reporting for specific materials, systems and methods

We require information from authors about some types of materials, experimental systems and methods used in many studies. Here, indicate whether each material, system or method listed is relevant to your study. If you are not sure if a list item applies to your research, read the appropriate section before selecting a response.

Materials & experimental systems

n/a	Involved in the study	
☒	☐	Antibodies
☒	☐	Eukaryotic cell lines
☒	☐	Palaeontology and archaeology
☐	☒	Animals and other organisms
☒	☐	Clinical data
☒	☐	Dual use research of concern
☒	☐	Plants

Methods

n/a	Involved in the study	
☒	☐	ChIP-seq
☒	☐	Flow cytometry
☒	☐	MRI-based neuroimaging

Animals and other research organisms

Policy information about studies involving animals; ARRIVE guidelines recommended for reporting animal research, and Sex and Gender in Research

Laboratory animals	The study did not involve laboratory animals.
Wild animals	Individuals of Acinonyx jubatus, Alces alces, Antidorcas marsupialis, Bison bison, Bison bonasus, Canis aureus, Canis latrans, Canis lupus, Capra ibex, Capreolus capreolus,Cervus elaphus, Chlorocebus pygerythrus, Crocuta crocuta, Cryptoprocta ferox, Equus hemionus, Erinaceus europaeus, Eulemur rufifrons, Felis chaus, Felis silvestris, Gazella subgutturosa, Genetta genetta, Ichneumia albicauda, Lepus europaeus, Lynx lynx, Lynx rufus, Madoqua kirkii, Odocoileus virginianus, Ovibos moschatus, Panthera leo, Panthera pardus, Papio anubis, Procyon lotor, Propithecus verreauxi, Puma concolor, Sus scrofa, Tragelaphus oryx, Tragelaphus strepsiceros, Ursus americanus, Ursus arctos, Viverra tangalunga, Vulpes bengalensis, Vulpes vulpes were fitted with GPS collars (A little backpack was fitted in the case of E. europaeus). For species-specific handling please see the respective study descriptions of the unique studies summarized in this study.
Reporting on sex	In the disturbance intensity model: Sex was included as a random smoothing effect, allowing for a smooth relationship between sex and the dependent variable. This allows for individual-specific effects of sex on the response, which can be useful when assuming that the relationship between sex and the response is not strictly linear but varies smoothly across individuals or species.<br>In the Recovery peed and duration models: Sex was included as independent variables.

Field-collected samples	The study did not involve samples collected from the field.
Ethics oversight	Acinonyx jubatus: All experimental procedures described were approved by the Internal Ethics Committee of the Leibniz Institute for Zoo and Wildlife Research (Leibniz-IZW, Berlin, Germany) (permit number: 1 April 2002) and the Ministry of Environment, Forestry and Tourism of Namibia (permit numbers: 1689/2012, 1813/2013, 1914/2014, 2067/2015, 2194/2016, 2208/2017, RCIV00082018/2018050101).<br>Alces alces: The capture and handling of GPS-marked moose in Norway was approved by the Norwegian Environment Agency (capture) and the Norwegian Food Safety Authority, which is the Norwegian animal research authority (permits no. 16/258650, 07/68902 and 2015/232016).<br>Antidorcas marsupialis: Approved by the Namibian Council on Research, Science and Technology, certificate: RCIV00032018.<br>Genetta genetta: Approved by The National Council of Science Technology and Innovation (permit number NACOSTI/P/14/357/2062), Kenya Wildlife Service (permit number KWS/BRM/5001) and through the Smithsonian Institution's National Museum of Natural History's Animal Care and Use Committee (Animal Study Proposal 2014-11).<br>Bison bison: Approved by the Nature Conservancy (Missouri) and Missouri Department of Natural Resources.<br>Bison bonasus: This study was carried out under research permits no. DLOPiK-op/ogiz-4200/IV.A-38-1/8310,10568/07/wo from the Polish Ministry of Environment and no. DOPozgiz-4200/IV.A-4/208/10/ls from the General Director for Environmental Protection in Poland, as well as ethics, permits no. 31/2006, and 2009/52 from the Local Ethical Commission in Białystok, Poland.<br>Canis aureus: All captures and handling were approved by the Maharashtra State Forest Department: permit no. SPP-147, dated 17.3.2015.<br>Canis latrans: Approved by Mississippi State University Institutional Animal Care and Use Committee, protocols 09-004, 12-012.<br>Canis lupus: Approved by Mississippi State University Institutional Animal Care and Use Committee, protocols 09-004, 12-012.<br>Capra ibex: The body in charge of the Alpine ibex capture was the Gran Paradiso National Park. Ibex capture and handling protocols were approved by the Italian Ministry of Environment (Protoc. no. 25114/04).<br>Capreolus capreolus: (IZW-Berlin) Approved by the ”LUGV Brandenburg”, permit: 23-2347-1-2009.<br>Capreolus capreolus: Game captures were conducted in accordance with European and French laws. The experiment was designed to minimize animal stress and handling time and to ensure animal welfare, as defined in guidelines for the ethical use of animals in research. A specific accreditation was also delivered to the OFB for capturing animals for scientific and wildlife management purposes. All methods were approved by the authorities (French Ministry of Environment). Roe deer captures and experimental procedures were in line with the French Environmental Code (Art.R421-15 to 421-31 and R422-92 to 422-94-1) and duly approved by legislation from the Prefecture of Paris (Prefectural Decree no. 2009-014).<br>Capreolus capreolus: All capture and marking procedures were done in accordance with French and European laws for animal welfare (prefectural order from the Toulouse Administrative Authority to capture and monitor wild roe deer and agreement no. A31113001 approved by the Departmental Authority of Population Protection).<br>Capreolus capreolus: Permit provided by the government of Upper Bavaria (ROB-55.2Vet-2532.Vet 02-17-190).<br>Capreolus capreolus: Resolution of the Provincial Government n. 602, under approval of the Wildlife Committee of 20/09/2011, and successive integration approved on the 23/04/2015.<br>Capreolus capreolus: The animal capture and handling protocols were authorized by the cantonal veterinary and animal welfare services with permit number BE75/11.<br>Cervus elaphus: Game captures were conducted in accordance with European and French laws. The experiment was designed to minimize animal stress and handling time and to ensure animal welfare, as defined in guidelines for the ethical use of animals in research. A specific accreditation was also delivered to the OFB for capturing animals for scientific and wildlife management purposes. Red deer captures, and experimental procedures were in line with the French Environmental Code (Art.R421-15 to 421-31 and R422-92 to 422-94-1) and duly approved by legislation from the Prefecture of Paris (Prefectural Decree no. 2009-014 and no. 2015-020).<br>Cervus elaphus: Permit provided by the Ministry of the Environment of the Czech Republic, number MZP/2019/630/361.<br>Cervus elaphus: Permit provided by the government of Upper Bavaria (Az. 55.2-1-54-2531-89-09).<br>Chlorocebus pygerythrus: The study was conducted with permission from the Kenya Government (NACOSTI permit no. P/15/5820/4650) and under IACUC protocol no. 17477 from the University of California, Davis.<br>Crocuta crocuta: Animal handling protocols were approved and conducted with the ethical clearance of the Animal Research Ethics Committee of the University of KwaZulu-Natal, South Africa (009/13/Animal), and the Institutional Animal Care and Use Committee of University of California at Berkeley (IACUC Protocol #R217-0512B) and Virginia Tech (IACUC Protocol #15-012). Scientific collecting permits were authorized from the Ministry of Environment and Tourism, Namibia (Research/Collecting Permits 1724/2012, 1834/2013, 1956/2014) and from the Department of Wildlife and National Parks, Botswana (Research Permit EWT 8/36/4 XXVIII [35]).<br>Cryptoprocta ferox: All research protocols were approved by the appropriate animal Use and care committees of Germany (”Bundesministerium fur Naturschutz, BfN”) and Madagascar (”Ministiere de l'Environnement et des Eaux et Forets, MINEEF”).<br>Equus hemionus: Animal tracking permit provided by the Ministry of Environment and Tourism, Mongolia.<br>Erinaceus europaeus: (IZW-Berlin) Approved by ethical standards of the institution (IZW permit 2016-02-01), German law ”Tierversuchsgenehmigung” permission numbers: Reg0115/15, and G0104/14, and the local nature conservation authority.<br>Eulemur rufifrons: All research protocols were approved by the appropriate Animal Use and Care Committees of Madagascar (Ministere de l'Environnement et des Eaux et For` ets, MINEEF: No 90/16/MEEMF/SG/DGF/DAPT/SCBT.RE,ˆ No 72/17/MEEMF/SG/DGF/DSAP/SCBT.RE.<br>Felis chaus: All captures and handling were approved by the Maharashtra State Forest Department: permit no. SPP-147, dated 17.3.2015.<br>Felis silvestris: Approved by the local nature conservation authority, permit number: ASTURIAS 2018/002528 LEON EP/CYL/666/2018.<br>Gazella subgutturosa: Animal tracking permit provided by the Ministry of Environment and Tourism, Mongolia.<br>Genetta genetta: Approved by The National Council of Science Technology and Innovation (permit number NACOSTI/P/14/357/2062), Kenya Wildlife Service (permit number KWS/BRM/5001) and through the Smithsonian Institution's National Museum of Natural History's Animal Care and Use Committee (Animal Study Proposal 2014-11).<br>Ichneumia albicauda: Approved by The National Council of Science Technology and Innovation (permit number NACOSTI/P/14/357/2062), Kenya Wildlife Service (permit number KWS/BRM/5001) and through the Smithsonian Institution's National Museum of Natural History's Animal Care and Use Committee (Animal Study Proposal 2014-11).

Lepus europaeus: Animal tracking was obtained in accordance with the Federal Nature Conservation Act (§ 45 Abs. 7 Nr. 3) and approved by the local nature conservation authority (reference numbers: 2347-6-2019, LUGV V3- 2347-22-2013, and 55.2-1-54-2532-229-13).

Lynx lynx: Approved by the PLA Moravian Karst Administration and the Czech Ministry of Environment, permit numbers: SR/0081/JM/2017; 34128/ENV/17-2146/630/17). Lynx live-trapping in Poland was approved by the National Ethics Committee for Animal Experiments (no. DB/KKE/PL—110/2001) and the Local Ethics Committee for Animal Experiments at the Medical University of Białystok, Poland (no. 52/2007).

Lynx lynx: Permit provided by the government of Upper Bavaria (Az. 55.2-1-54-2531-89-09).

Lynx rufus: All animal capture, handling, collaring, and sample collection was approved by the Institutional Animal Care and Use Committee (IACUC) of the University of California, Santa Cruz (Protocols "Seril 1701", and "Seril 1701 a1"). Scientific collecting permits were authorized by the California Department of Fish and Wildlife (Aromas, SCP-11968; Coyote Valley, SCP-13565).

Lynx rufus: Approved by Mississippi State University Institutional Animal Care and Use Committee, protocols 09-004, 12-012.

Madoqua kirkii: The research permit was approved by Mpala Research Center, Laikipia, Kenya.

Odocoileus virginianus: All activities were conducted according to guidelines established by the American Society of Mammalogists, and with authorization from the Oklahoma Department of Wildlife Conservation.

Ovibos moschatus: The study was approved by the Government of Greenland (permit no. 2019-88).

Panthera leo: Animal handling protocols were approved and conducted with the ethical clearance of the Animal Research Ethics Committee of the University of KwaZulu-Natal, South Africa (009/13/Animal), and the Institutional Animal Care and Use Committee of University of California at Berkeley (IACUC Protocol #R217-0512B) and Virginia Tech (IACUC Protocol #15-012). Scientific collecting permits were authorized from the Ministry of Environment and Tourism, Namibia (Research/Collecting Permits 1724/2012, 1834/2013, 1956/2014) and from the Department of Wildlife and National Parks, Botswana (Research Permit EWT 8/36/4 XXVIII [35]).

Panthera pardus: The study was conducted with permission from the Kenya Government (NACOSTI permit no. P/15/5820/4650) and under IACUC protocol no. 17477 from the University of California, Davis.

Papio anubis: The study was conducted with permission from the Kenya Government (NACOSTI permit no. P/15/5820/4650) and under IACUC protocol no. 17477 from the University of California, Davis.

Propithecus verreauxi: All research protocols were approved by the appropriate Animal Use and Care Committees of Madagascar (Ministere de l'Environnement et des Eaux et For` ets, MINEEF: No 90/16/MEEMF/SG/DGF/ˆ DAPT/SCBT.RE, No 72/17/MEEMF/SG/DGF/DSAP/SCBT.RE.

Procyon lotor: Approved by the "LUGV", permit number: 2347-7-2020.

Puma concolor: Research was approved by UCSC IACUC, proposal code Wilmc1312, and conducted under permit #11968.

Sus scrofa: All activities were conducted according to guidelines established by the American Society of Mammalogists; as defined by the Oklahoma Feral Swine Control Act (O.S § 6-601), only Judas pigs with tracking collars were released, all others were euthanized by law.

Sus scrofa: Approved by the ethics committee of the Ministry of the Environment Czech Republic number MZP/2019/630/361.

Sus scrofa: Approved by the Tuscany Regional Administration (permit number 103/5936/152 - 13/03/2002) and the Arezzo Province Administration (permit number 144160/42-41-2013 30/07/2013).

Sus scrofa: Approved by the Foreste Casentinesi National Park, permit numbers 626 - 10/12/2015 and 969 - 8/12/2018. Sus scrofa: Approved by Regione Autonoma della Sardegna, permit number: 4753-74 del 07/03/2017.

Sus scrofa: Approved by regional council Tubingen, according to animal welfare law¨ § 8.1 of the Federal State Baden-Wurttemberg, permit number: WFS1/12.

Tragelaphus oryx: Approved by the Namibian Council on Research, Science and Technology, certificate: RCIV00032018.

Tragelaphus strepsiceros: Approved by the Namibian Council on Research, Science and Technology, certificate: RCIV00032018.

Ursus americanus: Approved by Mississippi State University Institutional Animal Care and Use Committee, protocols 09-004, 12-012.

Ursus arctos: All captures and handling were approved by the Polish authorities (no. DOPOZ.6401.08.2.2013.ls, DOP-OZ.6401.08.2.2013.ls.1, DZP-WG.6401.08.8.2014.JRO), Polish ethical committee (no. 21/2013 and 101/2014) and Decree of Polish Ministry of Environment (Dz.Urz.M.S. 2017 poz. 2) and/or Slovak Ministry of Environment (MZP SR c.´ 3555/2012-2.2).

Viverra tangalunga: Approved by the Sabah Biodiversity Centre and the Sabah Wildlife Department, license ref.no: JKM/MBS.10000-2/2 JLD.6[8].

Vulpes bengalensis: All captures and handling were approved by the Maharashtra State Forest Department: permit no. SPP-147, dated 17.3.2015.

Vulpes vulpes: Approved by the Himachal Pradesh Forest Department: WLM/Research study/1259, dated 10/05/2019.

Vulpes vulpes: Approved by the "Landesamt fur Umwelt, Gesundheit und Verbraucherschutz Brandenburg" LUGV,¨ permit number: 2347-25-2015 and V3-2347 13-2011.

Vulpes vulpes: Approved by the animal welfare licensing committee of Berlin ("Landesamt fur Gesundheit und Soziales"¨ LaGeSo), permit number: G0211/15.

Note that full information on the approval of the study protocol must also be provided in the manuscript.

Plants

Seed stocks	*Report on the source of all seed stocks or other plant material used. If applicable, state the seed stock centre and catalogue number. If plant specimens were collected from the field, describe the collection location, date and sampling procedures.*
Novel plant genotypes	*Describe the methods by which all novel plant genotypes were produced. This includes those generated by transgenic approaches, gene editing, chemical/radiation-based mutagenesis and hybridization. For transgenic lines, describe the transformation method, the number of independent lines analyzed and the generation upon which experiments were performed. For gene-edited lines, describe the editor used, the endogenous sequence targeted for editing, the targeting guide RNA sequence (if applicable) and how the editor was applied.*
Authentication	*Describe any authentication procedures for each seed stock used or novel genotype generated. Describe any experiments used to assess the effect of a mutation and, where applicable, how potential secondary effects (e.g. second site T-DNA insertions, mosiacism, off-target gene editing) were examined.*
